# A Variable Genetic Architecture of Melanic Evolution in *Drosophila melanogaster*

**DOI:** 10.1534/genetics.116.192492

**Published:** 2016-09-15

**Authors:** Héloïse Bastide, Jeremy D. Lange, Justin B. Lack, Amir Yassin, John E. Pool

**Affiliations:** Laboratory of Genetics, University of Wisconsin–Madison, Wisconsin 53706

**Keywords:** *Drosophila melanogaster*, pigmentation, parallel evolution, bulk segregant analysis, standing genetic variation, adaptation

## Abstract

Unraveling the genetic architecture of adaptive phenotypic divergence is a fundamental quest in evolutionary biology. In *Drosophila melanogaster*, high-altitude melanism has evolved in separate mountain ranges in sub-Saharan Africa, potentially as an adaptation to UV intensity. We investigated the genetic basis of this melanism in three populations using a new bulk segregant analysis mapping method. We identified 19 distinct QTL regions from nine mapping crosses, with several QTL peaks overlapping between two or all populations, and yet different crosses involving the same melanic population commonly yielded distinct QTL. The strongest QTL often overlapped well-known pigmentation genes, but we typically did not find wide signals of genetic differentiation (*F_ST_*) between lightly and darkly pigmented populations at these genes. Instead, we found small numbers of highly differentiated SNPs at the probable causative genes. A simulation analysis showed that these patterns of polymorphism were consistent with selection on standing genetic variation. Overall, our results suggest that, even for potentially simpler traits like pigmentation, the complexity of adaptive trait evolution poses important challenges for QTL mapping and population genetic analysis.

IMPORTANT controversies persist regarding the process of adaptive trait evolution at the genetic level. First, phenotypic evolution may generally depend on “oligogenic” changes involving few loci or “polygenic” changes involving many loci (Bell 2009; Pritchard *et al.* 2010). Second, the molecular properties of beneficial mutations are debated, especially the relative importance of protein-coding *vs.* gene-regulatory changes (Hoekstra and Coyne 2007; Carroll 2008). Third, the contribution of standing genetic variation to adaptive change, relative to newly-occurring mutations, remains unresolved (Pritchard *et al.* 2010; Jensen 2014). A final question concerns the genetic predictability of adaptive trait evolution; when the same phenotype arises in two or more populations or species, how often does natural selection act on the same genes or even the same variants? Resolving these biologically important questions will require further empirical case studies pursuing the genetic basis of adaptive evolution.

Coloration has broad adaptive significance in survival and reproduction (Majerus 1998), making it an attractive target for genetic study. In most animals, melanin is synthesized by a small number of proteins whose patterning and sexual differentiation may be controlled by a myriad of transcription factors (Kronforst *et al.* 2012). In the fruit fly *Drosophila melanogaster*, wherein the melanin synthesis pathway is relatively clearly defined (Wittkopp *et al.* 2003; Massey and Wittkopp 2016), a recent study identified 28 *trans*-regulators of pigmentation (Rogers *et al.* 2014), and mutational screens have identified more than 400 genes that may impact body color (http://flybase.org). And yet, genome-wide association studies (GWAS) of female abdominal pigmentation variation (specifically measuring the black portion of the seventh, posterior-most abdominal segment) within four temperate populations all revealed major effects of the two melanin synthesis genes *tan* and *ebony* and the transcription factor *bric-a-brac 1* (*bab1*), although other minor effect genes were also detected (Bastide *et al.* 2013; Dembeck *et al.* 2015; Endler *et al.* 2016).

Pigmentation displays strong geographic trends in *D. melanogaster*. Several studies have reported a trend of darker cuticle at high latitudes in non-African populations, particularly with regard to the intensity of the thoracic trident (David *et al.* 1985; Das 1995; Telonis-Scott *et al.* 2011) and the width of black abdominal stripes (Munjal *et al.* 1997). An enrichment of pigmentation genes in genomic windows differentiating northern and southern populations was detected in a genome-wide selection scan in Australia (Reinhardt *et al.* 2014), paralleling the cuticular pigmentation cline found on this continent (Telonis-Scott *et al.* 2011). In tropical Africa, which harbors the ancestral range of the species (David and Capy 1988; Pool *et al.* 2012), unusually dark populations have been discovered in different mountain ranges (Pool and Aquadro 2007; Bastide *et al.* 2014). Overall, the pigmentation of African *D. melanogaster* is best predicted by UV intensity, offering a plausible selective agent to drive the recurrent evolution of melanism (Bastide *et al.* 2014). In one population (Uganda), a haplotype carrying a series of causative *cis*-regulatory mutations at *ebony* was a major contributor to melanism (specifically the background color of the fourth abdominal segment) and showed evidence of a strong partial selective sweep (Pool and Aquadro 2007; Rebeiz *et al.* 2009). However, no genome-wide search for loci underlying this parallel color evolution has been conducted.

Here, we investigate the genetic basis of melanic flies in three populations from Ethiopia, Cameroon, and Uganda, presenting the first application of a bulk segregant analysis (BSA) approach designed for *Drosophila* (Pool 2016). Many of the identified QTL contained major melanin synthesis genes. Some overlapping QTL between populations were detected, but differing sets of QTL were often detected between crosses from the same melanic population. Genetic differentiation at one locus (*ebony* in Ethiopia) was found to be consistent with natural selection acting on standing genetic variation.

## Materials and Methods

### Natural populations investigated

**Figure 1:**
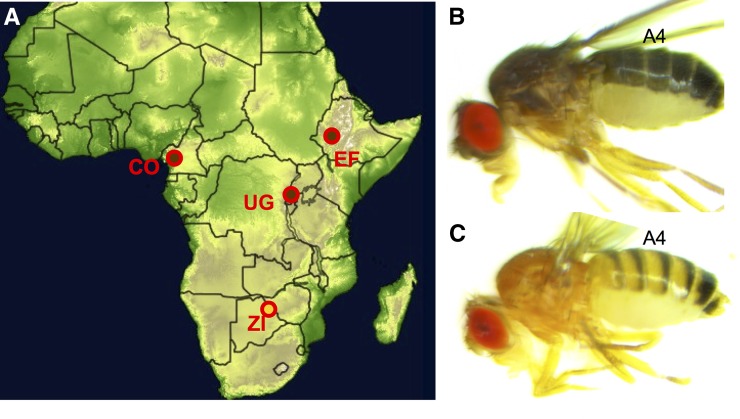
Populations sampled and studied phenotypes. (A) Several lines of each melanic population (brown and red circle; CO = Cameroon, EF = Ethiopia, and UG = Uganda) were separately crossed with homokaryotypic lines from a lightly pigmented population (yellow and red circle; ZI = Zambia). (B and C) Pigmentation phenotypes in Ethiopia (B) and Zambia (C) showing the fourth abdominal segment that was analyzed for mapping.

The populations in the present study were all studied by Bastide *et al.* (2014), where a number of individuals coming from 30 natural populations of various latitudes and altitudes were scored for body pigmentation. Among those, three Afrotropical populations showed an outstanding dark pigmentation and were used to found experimental crosses: Fiche, Ethiopia [EF, 9.81°N, 38.63°E, altitude (alt.) 3070 m] showing the most extreme phenotype with the entire body of the fly strongly melanized; Oku, Cameroon (CO, 6.25°N, 10.43°E, alt. 2169 m); and Namulonge, Uganda (UG, 0.53°N, 32.60°E, alt. 1134 m). In addition, a population from Siavonga, Zambia (ZI, 16.54°S, 28.72°E, alt. 530 m) was chosen as the reference light population against which all of the dark populations were subsequently crossed (Figure 1).

### Choice of parental lines for mapping crosses

We set four, three, and two experimental crosses each seeded with F_1_ flies produced from a cross between a darkly pigmented EF, CO, or UG line and a lightly pigmented ZI line, respectively (Supplemental Material, Table S1). All parental lines had been inbred for eight generations. Each mapping cross between a melanic population and Zambia involved distinct inbred lines (*e.g.*, EF1 × ZI1, EF2 × ZI2, *etc*.). To choose the parental lines, we tested lines from each population for the presence of eight common chromosomal inversions by PCR using primers and amplification conditions from Lack *et al.* (2016), to avoid seeding a cross with heterokaryotypic flies unable to recombine near the inversion. These included six autosomal cosmopolitan inversions (*In(2L)t*, *In(2R)NS*, *In(3L)P*, *In(3R)P*, *In(3R)K*, and *In(3R)Mo*), and two sub-Saharan inversions on the X chromosome (*In(1)A* and *In(1)Be*). We also used the pigmentation score data of the fourth abdominal segment of females that we generated in Bastide *et al.* (2014) to select darker lines for each melanic population (in conjunction with other phenotypes of interest).

### Experimental design for mapping crosses

We followed the experimental design described in Pool 2016. In this BSA approach to QTL mapping, a cross between two phenotypically-contrasting inbred strains is followed by multiple generations of interbreeding, and phenotypic selection only in the final generation. For each cross, we conducted reciprocal crosses between eight melanic strain flies and eight ZI strain flies, independently. From each of these two crosses, 125 random F_1_ males and 125 random F_1_ females were mixed together (*N* = 500). After combining the F_1_ flies from both reciprocal crosses, offspring were allowed to interbreed for 20 nonoverlapping generations at a population size of ∼1500 individuals in 28 × 14 × 15-cm plastic cages, each provided with 14 vials with standard *Drosophila* medium (containing molasses, corn meal, yeast, agar, and antimicrobial agents) and kept at ∼20°. Each generation, adults were allowed to lay eggs on the food for 1 week and then discarded, and food vials were replaced when the first new adult flies in the cage were 7–10 days old. After 20 generations (allowing a large number of unique recombination events to take place), we allowed adult flies to lay eggs on fresh food for 2 days before replacing the vials and waiting for F_20_ flies to emerge. We then visually phenotyped 3–5-day-old females (*N* = 600) for pigmentation on the fourth abdominal segment under CO_2_ anesthesia, focusing on either pigmentation intensity near the anterior margin (A4 background; crosses EB1, EB2, and UB1) or the width of the posterior black stripe (A4 stripe width; crosses CS1, CS2, CS3, ES1, ES2, and US1), as in Bastide *et al.* (2014). For each cross, flies were sorted into the 10% darkest (*N* = 60) and the 10% lightest (*N* = 60) females.

### Preparation of genome libraries

Genome libraries were independently prepared for the parental lines and the four F_20_ pigmentation subgroups for each cross. For each library, genomic DNA was extracted from a pool of 30 females by chloroform extraction and ethanol precipitation. DNA was then fragmented using the Bioruptor sonicator (Diagenode), and paired-end libraries with approximately 300-bp inserts were prepared using the NEBNext DNA Library Prep Reagent Set for Illumina (New England Biolabs no. E6000L). Library concentration and quality were assessed using an Agilent 2100 Bioanalyzer (Agilent Technologies) and were sequenced at the UW–Madison Biotechnology Center on the Illumina HiSequation 2000 platform with 100-bp paired read lengths.

### Alignment of the raw sequences

Reads were mapped to the *D. melanogaster* reference genome (release 5.57) using default parameters in BWA v0.6.2-r126 (Li and Durbin 2009). The BAM files were remapped with Stampy v1.0.21 (Lunter and Goodson 2011), and the reads were filtered for a mapping quality of 20 and for proper pairs with samtools v0.1.18 (Li *et al.* 2009). BAM files were cleaned by removing unmapped reads and sorted by coordinate, and PCR duplicates were marked using Picard v1.109 (http://picard.sourceforge.net). Alignment around indels was then improved using GATK v3.2 (McKenna *et al.* 2010; DePristo *et al.* 2011). Sequencing depth obtained for each mapping population sequencing is given in Table S1.

### Genome mapping of pigmentation genes using ancestry analysis

For each cross, we used the PoPoolation2 ver. 1.201 software package (Kofler *et al.* 2011) to generate a synchronized mpileup file for the two parental genomes and the pigmentation-sorted pools aligned to the *D. melanogaster* reference. For each biallelic SNP, an ancestry difference value (*a_d_*) summarized the difference in parental strain ancestry between the high and low phenotypic pools. With respect to the melanic parental line allele, *a_d_* was estimated as

$$a_{d}=\left(f_{H}-f_{L}\right)/\left(p_{H}-p_{L}\right)\text{,}$$

where *p_H_* is the frequency of the major allele in the melanic parental line, *p_L_* the frequency of the melanic allele in the nonmelanic parental line, *f_H_* the frequency of the melanic allele in the F_20_ dark subgroup, and *f_L_* is the frequency of the melanic allele in the F_20_ light subgroup. Only sites with parental strain frequency difference *p_H_ – p_L_* ≥ 0.25 were considered. The five chromosomal arms (X, 2L, 2R, 3L, and 3R) were subdivided into 2728, 3131, 2357, 2956, and 2935 windows, each of roughly 8.4-kb on average, whose boundaries were determined according SNP density in ZI genomes (Lack *et al.* 2015). For each cross, ancestry difference values were averaged across qualifying SNPs for each window. Scripts used in the preparation of mapping data can be found at: http://github.com/JohnEPool/SIBSAM1.

QTL mapping was performed using Simulation-based Inference for Bulk Segregation Analysis Mapping (SIBSAM; Pool 2016). Unlike BSA in yeast, where millions of segregants can be generated, BSA using *Drosophila* may often generate overlapping QTL peaks, which most BSA mapping approaches are not designed to account for. SIBSAM therefore analyzes both primary QTL peaks (the maximum value in an interval of continuously positive *a_d_*) and secondary QTL peaks that may flank them (Pool 2016). Simulations are conducted based on the full experimental process (with recombination in multiple females for multiple generations), selection on phenotype in the final generation (which is based on diploid genotype at each QTL, assuming additivity, plus environmental/measurement variance), followed by the sampling of sequence reads to obtain *a_d_*. To summarize trends across windows, we performed a simple smoothing of empirical and simulated *a_d_* values. We weighted the focal window’s value with a factor of five, and gave descending weights of four, three, two, and one for the four windows on each side.

SIBSAM involves a three phase inference process that results in estimates of the significance, location, and strength of QTL. First, simulations under the null model (no true QTL) are conducted to assess the significance of primary peaks. Peaks with at least a 95% true positive probability are carried forward in the analysis. For each of these significant primary peaks, simulations with a single QTL are conducted, with rejection sampling conditioned on the peak’s *a_d_* height yielding confidence intervals (CIs) for its effect size (the proportion of phenotypic variance explained) and genomic location. Empirically-observed secondary peaks are also compared against these single QTL simulations, asking how often the observed “secondary deviation” (the increase in *a_d_* between a local valley and a local peak) is generated when no secondary QTL is actually present. That analysis yields a *P*-value for each secondary peak (the null probability that the associated primary QTL would generate the observed secondary deviation by chance). If significant secondary peaks are present, simulations of multiple linked QTL are conducted. Rejection sampling conditioned on each peak’s height is used to refine the strength and location estimates of the primary and secondary peaks while accounting for these linked peaks’ effects on each other’s *a_d_* values (Pool 2016).

### Scan of genetic differentiation

To test whether significant QTL contained highly differentiated windows between the two populations from which the parental lines were drawn, we estimated *F_ST_* for these windows between Zambia and the melanic population in question. Genomes from ZI (*n* = 197), CO (*n* = 10), EF (*n* = 68), and Uganda (*n* = 40) were obtained from the *Drosophila* Genome Nexus (Lack *et al.* 2015). The Uganda population consisted of a pool of lines from Rwanda and Uganda that show minimal genetic differentiation (*F_ST_* = 0.015; Pool *et al.* 2012) and similarly dark pigmentation (Bastide *et al.* 2014). For each window, we also estimated the quantile (*Q*) of the window *F_ST_* relative to the empirical distribution of *F_ST_* of all windows per chromosomal arm, where *Q* denotes the proportion of windows with an equal or greater *F_ST_* value. Strong selective sweeps from new mutations are expected to produce high window-based *F_ST_* values due to hitchhiking effect, but such outliers may not include soft sweeps targeting alleles that already occur on multiple haplotypes; Lange and Pool 2016). Consequently, we also estimated the maximum SNP-based *F_ST_* in each window and the quantile of this value according to the empirical distribution of this estimate for all windows per chromosomal arm.

### Estimating a neutral null model for the Ethiopian population

The EF population harbors flies with some of the darkest phenotypes among all *D. melanogaster* populations (Bastide *et al.* 2014). Because the melanin synthesis gene *ebony* has previously been shown to be implicated in melanism in non-Ethiopian African populations (Pool and Aquadro 2007; Rebeiz *et al.* 2009) and was also found in our mapping analyses for Ethiopia, we compared its observed polymorphism to that expected under different selection scenarios. To estimate a neutral model consistent with the genetic diversity of this population, we used the allele frequency spectrum (AFS) from 105,715 SNPs from the EF, Rwanda (RG), and ZI populations, respectively, falling within autosomal short intronic segments (< 86-bp, with 16- and 6-bp removed from the intron start and end, respectively). These sites are presumably neutral (Halligan and Keightley 2006), although they can be affected by selection at linked sites. We then fitted our observed AFS of the three populations to the implemented “out of Africa” demographic model using a diffusion approximation approach implemented in the δaδi ver. 1.7 software package (Gutenkunst *et al.* 2009). This model (Figure S1) implies an instantaneous growth of the ancestral population (ZI) prior to an initial split, with a bottleneck taking place in the ancestor of the RG and EF populations. A second split between the two derived populations takes place followed by a bottleneck and a growth phase in each descendant population, with ongoing migration occurring among all populations. Parameters were optimized using δαδι and several runs were conducted to confirm that convergent parameter estimates were obtained. We then used nonparametric bootstrapping to infer parameter uncertainties. For this, we generated 100 bootstrapped AFS from the empirical data, and parameter SD were estimated using the Godambe Information Matrix (GIM) approach as implemented in δaδi version 1.7. Ancestral effective population size (*N_e_*) was inferred by dividing δaδi-estimated Watterson’s θ over four times the mutation rate, using an estimate from *D. melanogaster* of 3.27 × 10^−9^ (Schrider *et al.* 2013). Divergence times (in years) were then estimated assuming 15 generations per year (Pool 2015).

### Simulations

We used *msms* (Ewing and Hermisson 2010) to simulate a region motivated by the *ebony* locus. The optimized demographic parameters from the δaδi analysis used in these simulations were converted from units of 2*N_e_* to 4*N_e_* generations. Recombination rates were based on the local estimate by Comeron *et al.* (2012). The empirical data that we sought to emulate came from a 5001-bp window centered on the most differentiated SNP. At this site, the empirical data included 33 genomes from Ethiopia EF and 189 from Zambia ZI. Our simulations matched these sample sizes while also simulating a third population, representing Rwanda RG, which was present in the demographic modeling and simulations, but not included in the analysis of simulated data. Examining a range of scenarios with and without positive selection, we examined the propensity of each simulation to generate the disparate window *F_ST_* and maximum SNP *F_ST_* values observed between the EF and ZI samples at *ebony*.

We first ran 10,000 neutral simulations to test whether the values observed at *ebony* were unexpected in the absence of selection. We then explored a range of scenarios with positive selection limited to the Ethiopian population. Here, we varied the selection coefficient (*s*) and the frequency of the favored allele at the start of the sweep. In these simulations, we explored the hypothesis that the most differentiated SNP observed at *ebony* was a beneficial mutation (in Ethiopia only), and we set the target of selection to be the middle of the simulated 5001-bp locus. We simulated 10 different selection strengths and 6 starting beneficial allele frequencies for a total of 60 scenarios of selection. The 10 selection coefficients we studied were 0.00005, 0.000075, 0.0001, 0.0025, 0.005, 0.0075, 0.001, 0.0025, 0.005, and 0.0075. The six starting beneficial allele frequencies were 1/2*N*_e_, 0.005, 0.01, 0.025, 0.05, and 0.1.

In these simulations, our primary interest was to ask which models could replicate the observed maximal SNP frequency difference while generating a window *F_ST_* as low as that observed at *ebony*. To emulate the observed SNP frequencies in simulations with selection, we employed a subsampling strategy. We initially simulated larger samples of chromosomes for the Zambia (*n* = 250) and Ethiopia (*n* = 50) populations, then pared them down to 189 and 33 chromosomes, respectively. Subsampling was then pursued to achieve a nontrivial probability of a simulation matching our observed allele counts in both populations. Simulations lacking sufficient numbers of both alleles in either population to achieve these subsamples were rejected. Although we used the –SFC flag of *msms* to condition on the beneficial allele’s persistence in the Ethiopian population at the time of sampling, simulations could still be rejected if this allele was unsampled or sampled at low frequency. For each selection scenario, we ran a maximum of 2,500,000 simulations in an attempt to get 1000 accepted simulations.

We also filtered simulations to clearly distinguish between the predictions of hard sweeps and soft sweeps. For the case of an initial beneficial allele frequency of 1/2*N*_e_ (corresponding to new mutations), only hard sweeps could be generated. For all other initial frequencies, we required at least two unique copies of the beneficial allele to be present at sampling, to study soft sweeps specifically.

### Data availability

Raw sequence data are available from the NIH Short Read Archive with accession numbers given in Table S1.

## Results

### Large effect loci underlie melanism in the three populations

We used a newly-developed BSA method (Pool 2016) to map pigmentation QTL, *i.e.*, SIBSAM. We performed nine mapping crosses, each pairing an inbred line from one of our melanic populations (Cameroon, Ethiopia, or Uganda) with an inbred line from a lightly pigmented ancestral range population (Zambia). Mapping focused on two female pigmentation traits, A4 abdominal background color and A4 stripe width. For each cross, offspring interbred for 20 generations at a population size of ∼1500. In the final generation, the 10% darkest and lightest flies among 600 scored females were collected and sequenced as bulk pools for each cross.

A key goal of SIBSAM is to distinguish two or more linked QTL with overlapping mapping signals, which may occur when complex traits are mapped on this experimental scale. SIBSAM uses detailed experimental simulations in a three phase approach to make QTL inferences (*Materials and Methods*; Pool 2016). For each QTL peak, this process returns its estimated false positive probability, effect size estimate, C.I. (in terms of the proportion of a mapping cross’s phenotypic variance explained), and genomic C.I. These estimates are obtained both for each primary QTL peak (the maximum point in the interval of a contiguous QTL mapping signal) and for each secondary peak (lesser local maximum within such an interval; Pool 2016).

**Figure 2:**
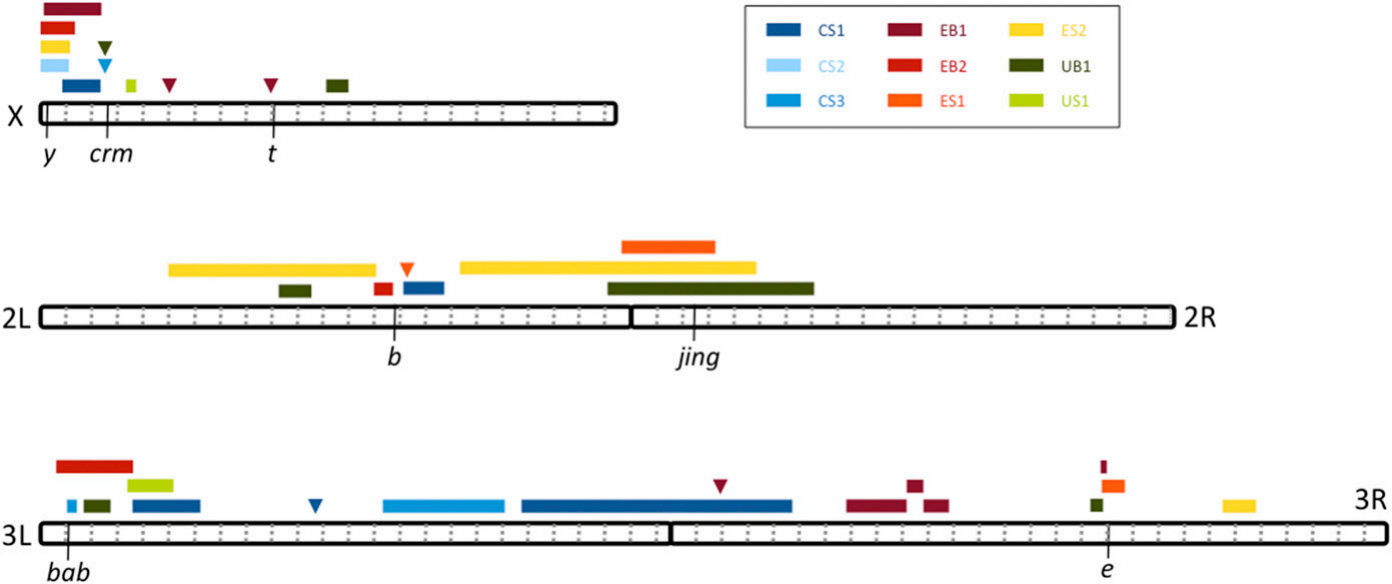
Locations of the detected QTL are shown with respect to the five major euchromatic chromosome arms of *D. melanogaster*. Colors indicate distinct crosses involving Cameroon (C), Ethiopia (E), and Uganda (U), mapping either background color (B) or stripe width (S) for the fourth abdominal segment of females. Boxes indicate 90% C.I. of each QTL, except that QTL intervals extending < 200-kb are marked with triangles. Dotted gray lines indicate Mb increments (for the release five genome) and black lines illustrate the positions of pigmentation candidate genes discussed in the text.

**Figure 3:**
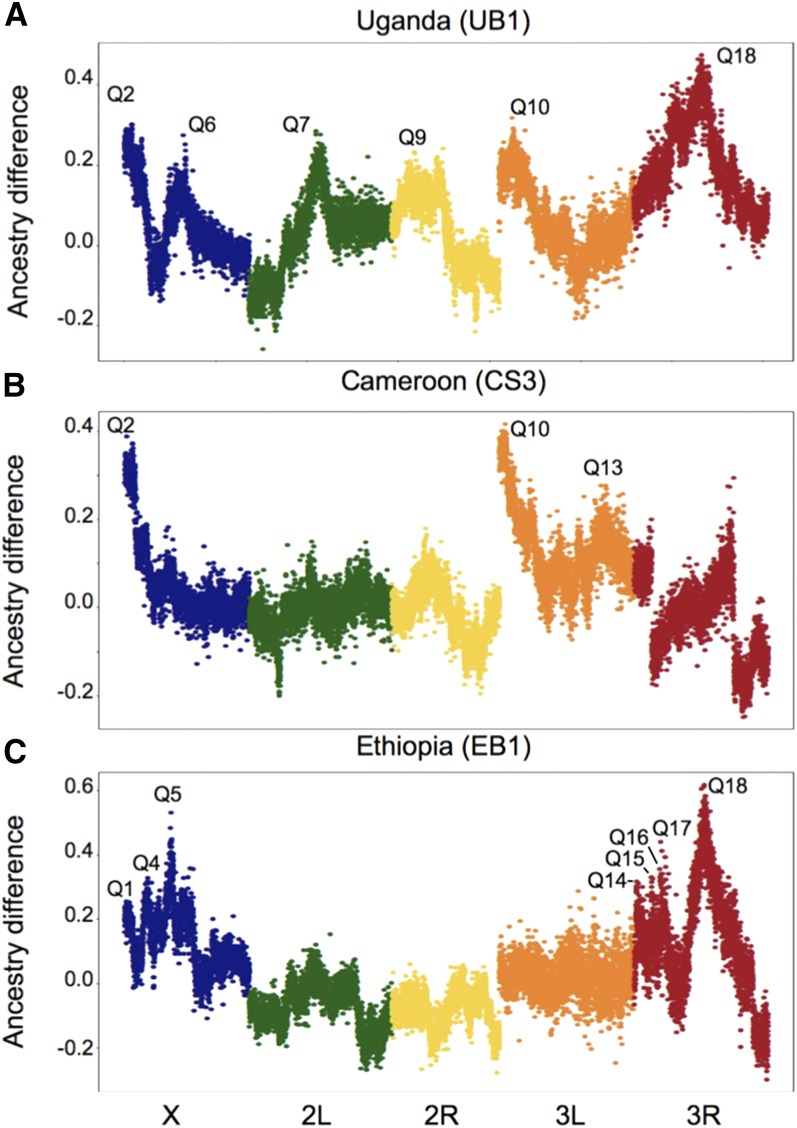
Ancestry difference plots showing relative proportions of the melanic parental population allele across five colored chromosomal arms (X, 2L, 2R, 3L, and 3R) in crosses involving three melanic populations crossed to the lightly-pigmented Zambia population: (A) Uganda (UB1), (B) Cameroon (CS3), and (C) Ethiopia (EB1). This raw mapping surface is an input for SIBSAM (Simulation-based Inference for Bulk Segregation Analysis Mapping). QTL names are according to Table 1. Discontinuities in the Cameroon plot’s chromosome arm 3R reflect the presence of *In(3R)K* in both parental strains, an inversion that is nearly fixed in the CO sample.

A total of 35 significant QTL peaks were found in the nine crosses (Figure 2, Figure 3, Figure S2, and Figure S3), each with a false positive probability below 5% (Table S2). Effect sizes ranged from 6.29 to 37.11% for primary peaks and from 5.66 to 25.92% for secondary peaks. Based on overlapping genomic confidence intervals, these can be summarized into 19 major QTL regions, ranging from 4-kb to 14-Mb long, of which 10 were unique to different single crosses (Table 1). Notably, even when the same trait was investigated in the same population, QTL often differed considerably between independent crosses. Many of these QTL peaks are tall enough that we expect very high power to detect them (Table S2; Pool 2016), suggesting that discordant results are unlikely to result solely from a randomly detected subset of shared QTL.

**Table 1:** QTL underlying melanic evolution identified from nine crosses between three high-altitude and one low-altitude sub-Saharan populations of *D. melanogaster*

QTL	Coordinates*^a^* (kb)	Candidate Genes*^b^*	Cameroon			Ethiopia				Uganda	
			CS1	CS2	CS3	EB1	EB2	ES1	ES2	UB1	US1
Q1	X:0–2424	*ewg*, *y*, *svr*, *CG13364*, *br*, *Rbcn-3B*, *Hr4*, *CG14054*, *CG14052*	***P***	***P***		S	***P***		***P***		
Q2	X:2519–2677	*per*, *Csat*, *crm*			***P***					***P***	
Q3	X:3367–3729										P
Q4	X:5327–5331					***S***					
Q5	X:9008–9016	*fend*, *t*, *dalao*				***P***					
Q6	X:11144–12007	*Gr10b*, *CG1749*, *FucT6*, *Amun*, *m*, *dy*, *ATP7*, *Usp7*								P	
Q7	2L:9302–10602	*Mco1*, *Cpr30B*, *CG5846*, *bib*, *Trp1*, *Cpr31A*, *CG13137*, *da*							P	P	
Q8	2L:12994–15773	*Vha68-1*, *CG9932*, *CG31849*, *CG9302*, *CG9008*, *Sos*, *b*, *CG16886*, *rk*, *CR31840*, *CG18095*, *nht*	P				***P***	***P***			
Q9	2L:16327–2R:7138	*CG42389*, *CG15143*, *Pde11*, *Mst36Fb*, *CG10348*, *Catsup*, *Rpn3*, *CG10492*, *Ddc*, *l(2)37Cc*, *l(2)37Cd*, *Aats-asn*, *CG10463*, *sick*, *CG31678*, *CG9336*, *CG3635*, *CG10395*, *laccase2*, *CG17508*, *CG7881*, *jing*, *Dhx15*, *p47*, *CG1620*, *CG8728*, *Dic3*, *Odc1*, *pdm3*, *CG30356*, *CG8083*, *CNT1*, *brp*, *Su(Var)2-10*, *unpg*, *lola*, *CG12309*						S	S	P	
Q10	3L:673–3383	*bab1*, *bab2*, *mwh*, *Cht2*, *mu2*, *SCOT*, *CG32301*, *CG1275*, *CG32298*, *CG16758*, *CG15812 (Pfk)*, *CG12010*			***P***		P			P	
Q11	3L:3383–6238	*scrt*, *CG15023*, *tgo*, *Membrin*, *CG10635*, *Txl*, *PVRAP*	P								P
Q12	3L:11672–11678		P								
Q13	3L:13344–18098	*tv*, *Tgi*, *CG9040*, *CG13481*, *Tdrd3*, *CG13461*, *Plp*, *CIC-c*, *aos*, *mbf1*, *Mo25*, *Baldspot*, *CG9715*, *CG13725*, *CG6479*, *Cad74A*, *Eip74EF*			P						
Q14	3L:18762–3R:4863	*CG8786*, *Mi-2*, *CG14182*, *gig*, *CG42674*, *CG6933*, *Spn77Bb*, *CG5955*, *CG12971*, *Hr78*, *VhaM9.7-b*, *CG43980*, *Vps11*, *CG12581*, *rtp*, *CG1113*, *Snr1*, *Ir84a*, *Atu*, *CG11373*, *Ir84a*, *dsx*, *Os-C*, *nac*, *Ctr1B*, *CG11753*, *M1BP*	P			S					
Q15	3R:6800–9200	*cu*, *ZnT86D*, *CG5214*, *Lk6*, *COX5A*, *dpr15*, *CG10013*, *MBD-R2*, *GstD3*, *CG6225*, *CG31347*, *CG8483*				S					
Q16	3R:9200–9860	*GILT1*, *CG14372*, *rdx*				S					
Q17	3R:9860–10830	*Npc2b*, *CG31321*, *ATPsynE*, *soti*, *cv-c*, *kibra*, *btsz*, *CG33332*, *VhaPPA1-2*, *VhaPPA1-1*				P					
Q18	3R:16329–17699	*bon*, *CG4000*, *Synd*, *CG5745*, *meigo*, *e*, *CG6475*				***P***		***P***		***P***	
Q19	3R:21485–22817	*tx*, *Pdf*, *Tb*, *Lerp*, *sda*, *CG31076*							***P***		

P, primary peak; ***P***, effect size of > 20%; S, secondary peak.

a Coordinates according to reference genome release five.

b In addition to well-studied pigmentation pathway genes and regulators, the listed genes include described *trans*-regulators of pigmentation (Rogers *et al.* 2014), genes detected in pigmentation genome-wide association studies (Dembeck *et al.* 2015), and genes with a mutant annotation of body color defective.

Among the common QTL peak regions, one (Q1) was shared among five crosses. This region consists of the 2.4-Mb telomeric end of chromosome X containing the important melanin synthesis gene *yellow*, which has been implicated in the evolution of pigmentation between multiple *Drosophila* species (Wittkopp *et al.* 2002; Jeong *et al.* 2006; Ordway *et al.* 2014). Four other QTL regions were detected in three unique crosses each. Three of these regions correspond to well-studied pigmentation genes. Q8 contains the melanin synthesis gene *black*, and it was shared among two Ethiopian and one Cameroonian crosses. To our knowledge, *black* has never been found to underlie *Drosophila* pigmentation evolution (Massey and Wittkopp 2016), although this enzyme constitutes an integral part of the *tan-ebony* loop (Wittkopp *et al.* 2003), the most evolutionarily labile part of the melanin synthesis network in *Drosophila*. The three QTL are all near *black*, but not all overlap it, a pattern that could reflect the contribution of other genes instead of *black*, or the chance exclusion of *black* from a QTL confidence interval, or an influence of undetected minor effect loci on QTL localization.

Among the other regions containing three QTL, Q9 is a broader region spanning the second chromosome centromere. The overlap between these QTL contains *jing*, which has been shown to affect abdominal pigmentation (Culi *et al.* 2006; Rogers *et al.* 2014) but has not been linked to pigmentation evolution. Q10 includes the transcription factor *bab1* and was shared by one cross from each of the three melanic populations. The other region (Q18) was shared by two crosses from Ethiopia and one cross from Uganda, and includes the melanin synthesis gene *ebony*. In Uganda, a strong effect of *ebony* on pigmentation has previously been illustrated (Pool and Aquadro 2007; Rebeiz *et al.* 2009), providing a “positive control” for our mapping method.

The remaining peaks did not include other major melanin synthesis enzymes (Table 1), with an exception in a single Ethiopian cross where a strong primary peak (Q5) included *tan*. Also notable was a pair of narrow QTL for Cameroon and Uganda (Q2) centered on *cramped*, which can influence abdominal pigmentation in a temperature-sensitive manner (Gibert *et al.* 2007, 2011). *bab1*, *ebony*, and *tan* have frequently been associated with pigmentation variation in cosmopolitan populations, while the other genes discussed here have not (Kopp *et al.* 2003; Bickel *et al.* 2011; Bastide *et al.* 2013; Rogers *et al.* 2013; Endler *et al.* 2016; Dembeck *et al.* 2015; Johnson *et al.* 2015; Miyagi *et al.* 2015). Many of the QTL regions not mentioned above contain one or more genes with a potential influence on pigmentation based on prior molecular, mutant, or association mapping studies (Table 1). However, three other regions contain no such gene, which might indicate that our list of pigmentation candidate genes is still not comprehensive.

### Low genetic differentiation at pigmentation-associated loci in Ethiopia

To investigate whether pigmentation-associated peaks harbor genes that have been strongly differentiated between the lowland ZI population and each of the melanic populations, we estimated their differences in allele frequencies using *F_ST_* for windows that have been used in the ancestry analyses (Table S3 and Table S4). While outliers for window *F_ST_* could indicate loci subject to population-specific natural selection, some of these may be unrelated to pigmentation evolution. In light of the strong trend toward major QTL occurring near well-known pigmentation genes, we discuss the genetic differentiation of these genes in particular, in addition to the full QTL regions that contain them.

**Figure 4:**
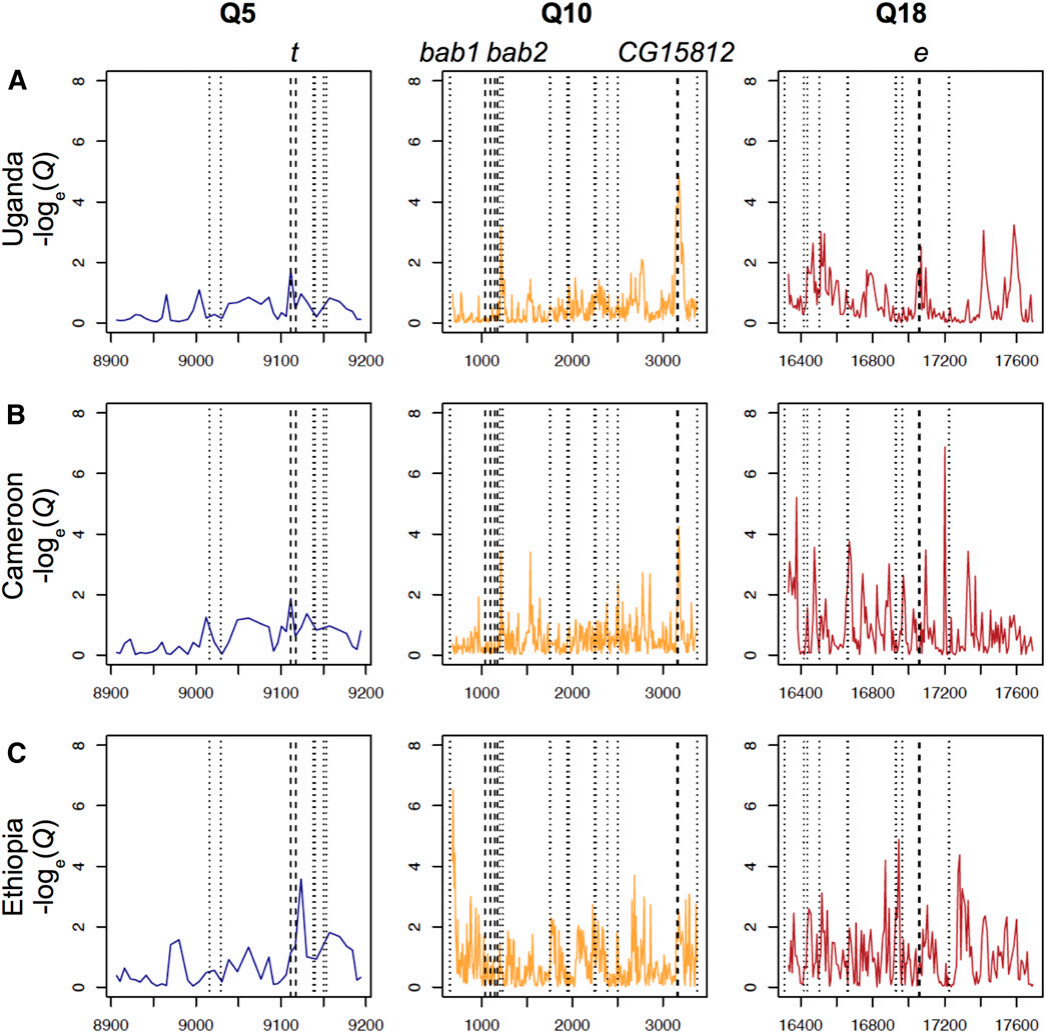
Window-based genetic differentiation (*F_ST_*) in quantiles (*Q*) between a lightly pigmented population (Zambia) and three melanic populations: (A) Uganda, (B) Cameroon, and (C) Ethiopia at three pigmentation-associated QTL. Dashed lines refer to boundaries of pigmentation candidate genes: *tan* (*t*), *bric-a-brac* (*bab1* and *bab2*), and *ebony* (*e*). Dotted lines represent the locations of other genes that may influence pigmentation (Table 1). Coordinates are given in kb with respect to release five of the *D. melanogaster* genome. In many cases, strong window genetic differentiation was not observed at pigmentation genes within large-effect QTL.

Consistent with previous findings of an incomplete selective sweep at the gene *ebony* in Uganda (Pool and Aquadro 2007; Rebeiz *et al.* 2009), window *F_ST_* between Uganda and Zambia detected a moderate peak around this locus, although these windows did not fall within the most extreme 5% (*i.e.*, *Q* > 0.05; Figure 4A). Among the remaining seven QTL in this population, three contained windows with elevated differentiation, including for the X-linked melanin synthesis gene *yellow* (Q1; *Q* < 0.001; Figure S4A); however, genetic differentiation tends to be broadly elevated across this low recombination telomeric region, so genetic variation at *yellow* should be interpreted with caution. Elevated *F_ST_* was also detected for a window upstream of the two transcription factor paralogs *bric-a-brac* (Q10; *Q* < 0.05; Figure 4A), and Q11 containing *CG15023* (*Q* < 0.01; Figure S4C), which may affect pigmentation (Mummery-Widmer *et al.* 2009).

In Cameroon, significant differentiation was found within five QTL detected in this population, including windows similar to that found in Uganda within Q1 (*Q* < 0.001; Figure S4B), Q10 (*Q* < 0.05; Figure 4B), and Q11 (*Q* < 0.05; Figure S4C). Three peaks of differentiation (*Q* < 0.05) were found at Q8 surrounding the melanin synthesis gene *black* but no peak at the gene itself (Figure S4B). None of these peaks contain any known pigmentation-related genes.

In Ethiopia, strong differentiation was found in all but 2 (Q10 and Q16) of its 12 QTL, but in general these peaks did not fall near pigmentation candidate genes. The four melanin synthesis genes (Figure 4C and Figure S4C) in the QTL of this population did not show strong signals of window genetic differentiation, including *ebony* in spite of the major effect of this QTL in the cross with the darkest phenotype.

### Strongly differentiated SNPs at candidate pigmentation genes in Ethiopia

Many of our stronger QTL overlapped a small handful of pigmentation genes that frequently underlie pigmentation variation and evolution (Table 1). And yet, variation at most of these genes did not show clear window-scale evidence of elevated genetic differentiation. Importantly, window-based *F_ST_* estimates are strongly influenced by the specific model of positive selection, with soft and/or incomplete sweeps potentially producing different degrees of window genetic differentiation than classic hard/complete sweeps (Lange and Pool 2016). In the case of an extreme soft sweep, in which a beneficial allele rises in frequency on many different haplotypes, elevated *F_ST_* might be limited to the causative variant and those very close to it.

**Figure 5:**
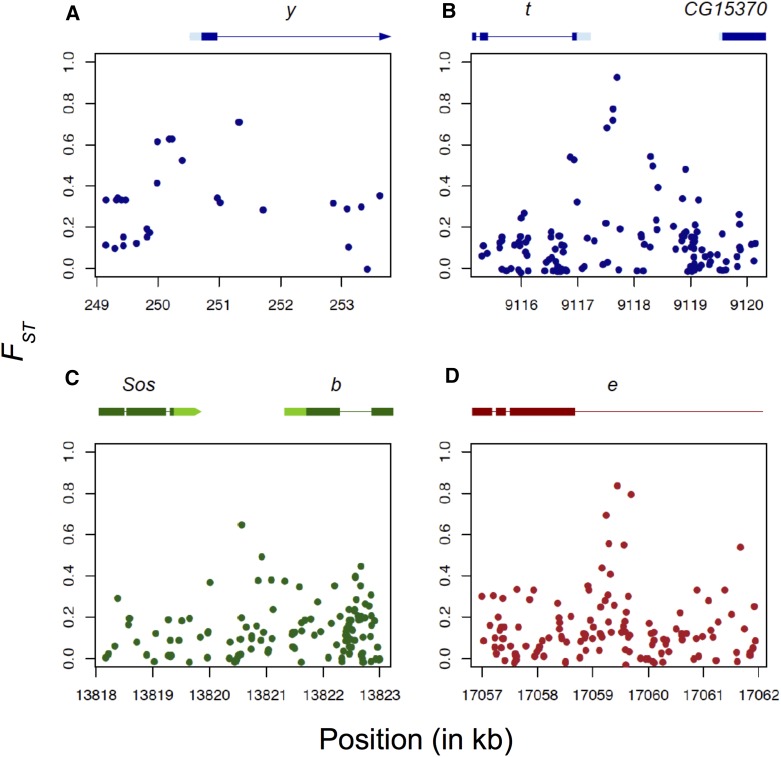
SNP-based genetic differentiation (*F_ST_*) estimates between lightly pigmented Zambia and darkly pigmented Ethiopia populations at four melanin synthesis enzyme genes: (A) *yellow* (*y*), (B) *tan* (*t*), (C) *black* (*b*), and (D) *ebony* (*e*). Each plot represents a 5-kb window centered on the most differentiated SNP for each gene. Lightly colored boxes refer to genes’ 5′ and 3′ UTRs, darkly colored boxes refer to exons, and lines refer to introns.

Therefore, we also investigated *F_ST_* values for individual SNPs at four candidate genes within QTL (*black*, *ebony*, *tan*, and *yellow*) for Ethiopia *vs.* Zambia. In spite of the lack of strong window *F_ST_* signal cited above, all four of these genes had one or more individual SNPs with strong differentiation, yet without any strong pattern of differentiation at linked SNPs (Figure 5). In each case, the SNP with maximal *F_ST_* had the derived allele at higher frequency in Ethiopia. Since individual SNP *F_ST_* may be more vulnerable to random variance when sample sizes are small, we note that for the SNPs described below, or sample sizes range from 33 to 55 genomes for Ethiopia, and from 188 to 191 for Zambia.

At *yellow* (Figure 5A), the most differentiated SNP (X:251,323; coordinates with respect to release 5 of the *D. melanogaster* reference genome) fell in the first intron. Although this region is not known to affect abdominal pigmentation in cosmopolitan *D. melanogaster*, in *D. pseudoobscura* and *D. virilis*, two species that are completely dark and whose phenotype resembles the dark Ethiopian phenotype, intronic enhancers affect body pigmentation (Kalay and Wittkopp 2010).

At *tan* (Figure 5B), the most differentiated SNP (X:9,117,695) did not fall in the male-specific-enhancer (*t_*MSE) which had often been detected in within-species studies focusing on the posterior abdominal segments in *Drosophila* (Bastide *et al.* 2013; Dembeck *et al.* 2015; Yassin *et al.* 2016) rather than nonsexually differentiated abdominal segments like this one. Instead, the most differentiated SNP fell 400-bp upstream of the beginning of the gene in a binding site of the transcription factor *dorsal* (The modENCODE Consortium *et al.* 2010), which is involved in the melanization defense response (Bettencourt *et al.* 2004). This SNP is tightly linked to a short indel 7-bp away. Nearby sequence closer to the promoter was recently shown to underlie thermal plasticity in pigmentation (Gibert *et al.* 2016).

At *black* (Figure 5C), the most differentiated SNP (2L:13,820,561) falls 700-bp upstream of the beginning of the gene in a region that was shown to bind with the homeotic transcription factor *prd* (The modENCODE Consortium *et al.* 2010). The enhancers of *black* affecting pigmentation in different body parts are yet to be studied.

At *ebony* (Figure 5D), the most differentiated SNP (3R:17,059,445) in Ethiopia falls within the first intron, which coregulates *ebony* expression in the abdomen together with an upstream enhancer known as the core abdominal *cis*-regulatory element (*e*_abdominalCRE; Rebeiz *et al.* 2009). In Uganda, a partial selective sweep was detected at the latter element (Pool and Aquadro 2007; Rebeiz *et al.* 2009). This element drives expression of *ebony* in the abdomen that is repressed by an unknown enhancer in the intron (Rebeiz *et al.* 2009).

### Soft selective sweeps can generate the pattern of polymorphism found at ebony

The recurring pattern of isolated SNPs with high *F_ST_* raises the question of whether these data result from genetic drift and sampling variance, weak hard sweeps, or soft sweeps. Therefore, we performed a simulation analysis to identify evolutionary scenarios that might be consistent with the differentiation patterns revealed at melanin synthesis genes in Ethiopia, *i.e.*, low window *F_ST_* across these gene regions, and yet small numbers of SNPs showing strong population frequency differences. We focused on the case of *ebony* in Ethiopia, where one SNP was found to have *F_ST_* = 0.85, but a 5-kb window around this site had an overall Ethiopia-Zambia *F_ST_* of just 0.17.

To identify a plausible null model, we used δaδi (Gutenkunst *et al.* 2009) to estimate demographic parameters under a three population model, using allele frequency data from the middles of autosomal short introns (Halligan and Keightley 2006), from our Ethiopia, Rwanda, and Zambia populations. Parameter estimates, as detailed in Table S5, entailed a recent split between the Ethiopia and Rwanda populations (roughly 1100 years ago, 95% C.I. 435–1765), with an initial bottleneck to ∼1% of the ancestral population size followed by exponential growth. Although we could not investigate all possible historical models, and the effects of natural selection on genomic variation could impact the precision of these estimates, the demographic estimates obtained here serve our primary objective of providing a neutral model capable of generating population genetic data resembling that of our empirical populations.

**Figure 6:**
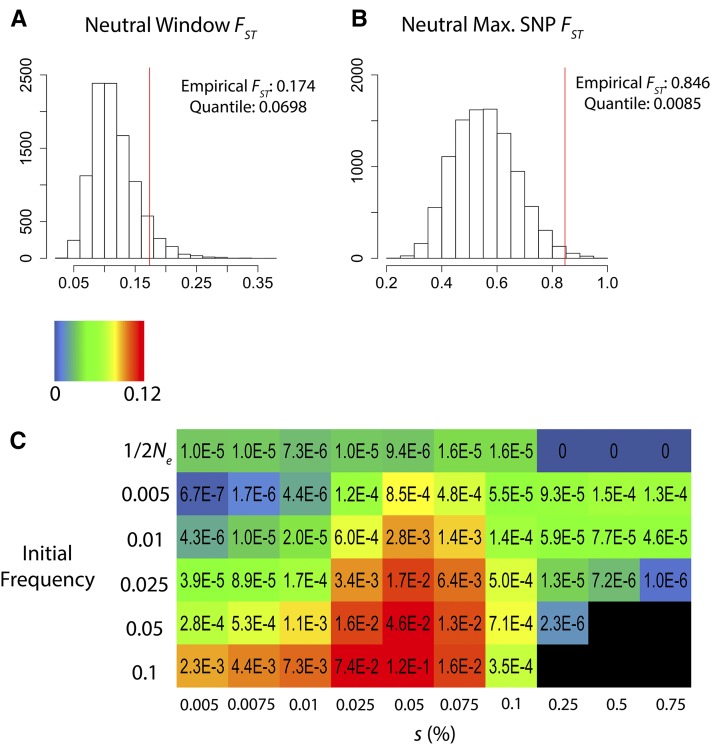
A simulation analysis was conducted to identify evolutionary models compatible with genetic differentiation at *ebony* between Ethiopia and Zambia populations. The top panels show that compared with neutral simulations, the empirically observed 5-kb window *F_ST_* is only moderately elevated (A), but the maximum SNP *F_ST_* observed at *ebony* is unusually high (B). (C) The heat map (C) illustrates outcomes of simulations in which the most differentiated SNP was favored in Ethiopia. The acceptance rates depicted here depend on: (1) population allele frequencies at the focal SNP that are compatible with subsampling to match empirical counts, and (2) a window *F_ST_* at least as low as that observed at *ebony*. Acceptance rates are colored based on a log_10_ scale, with black cells indicating < 10 successfully subsampled simulations out of 2.5 million. A range of selection strengths are depicted for models producing hard sweeps (initial frequency 1/2*N_e_*) and those conditioned on soft sweep outcomes (all others). Results suggest that soft sweep scenarios with higher initial frequencies are the most likely to raise the beneficial allele to high frequency (without fixing it), while also recapitulating the disparity between window *F_ST_* and SNP *F_ST_* observed at *ebony*.

We then generated neutral simulation data to study window *F_ST_* and maximum SNP *F_ST_* under this null model. Compared with neutral simulations based on this demographic model, we found that our window *F_ST_* observed at *ebony* was only marginally elevated (*Q* = 0.07), but maximum SNP *F_ST_* deviated more strongly from neutral predictions (*Q* < 0.01; Figure 6A). Thus, it is unlikely to observe this SNP frequency difference under the neutral model, and yet the window signal for *F_ST_* is probably insufficient to be detected in a typical genome scan.

Next, we conducted simulations based on the hypothesis that the most differentiated SNP at *ebony* was a target of positive selection in the Ethiopian population, varying the selection coefficient and the initial frequency of the beneficial allele. We rejected simulation replicates that could not be subsampled to match empirically observed allele counts at the target SNP (with 29 out of 33 Ethiopia genomes and 5 out of 189 Zambia genomes carrying the simulated beneficial allele, out of 50 and 250 total genomes simulated for the two populations, respectively). Thus, simulation replicates in which the beneficial allele was fixed or else failed to rise much in frequency were rejected. For each simulation scenario, we tested how often the SNP *F_ST_* entailed by the above counts was paired with a window *F_ST_* at least as low as we observed for *ebony*.

The scenarios that best matched the above frequency and *F_ST_* criteria were soft sweeps with higher initial frequencies (Figure 6B and Table S6). Weak hard sweeps could also match the *F_ST_* disparity, but these scenarios almost never allowed the beneficial allele to rise to high frequency on the time-scale of the Ethiopian population (Table S6). Hence, selection from standing genetic variation appears to be the strongest hypothesis for the patterns of genetic variation observed at *ebony* in the Ethiopian population.

A soft sweep is compatible with the presence of the “Ethiopian allele” at the focal SNP in Zambia at an estimated frequency of 2.6%. However, further study will be needed to test whether this putative selective event at *ebony* is indeed related to pigmentation evolution, and whether other *ebony* variants may contribute as well. Contributions of multiple SNPs or haplotypes at a locus have previously been described for *ebony* in Uganda (Rebeiz *et al.* 2009) and for toxin sensitivity in a cross-population mapping panel (Kislukhin *et al.* 2013; King *et al.* 2014).

## Discussion

We have integrated quantitative genetic and population genetic approaches to investigate the genetic basis of parallel melanism in three sub-Saharan populations of *D. melanogaster*. Our BSA approach revealed between 1 and 10 QTL per population/trait. The strongest QTL in each cross was estimated to explain 16–37% of the parental strain differences. Importantly, these effect size estimates can have upward bias in some scenarios (Pool 2016) and do not account for dominance or epistatic variance; these estimates should therefore be viewed as preliminary. Some QTL may have been missed by our study for a number of reasons: minor effect QTL may exist below the threshold of our approach, power to detect closely linked QTL may be limited (Pool 2016), and rare but strong QTL may have been absent from our parental strains by chance. Still, it seems clear that QTL of moderate to large effect contribute to melanic evolution in these populations. An important role for larger-effect QTL may be expected for locally adaptive traits subject to migration-selection balance (Yeaman and Whitlock 2011), in contrast to predictions for within-population variation under stabilizing selection (where weak and/or rare effects may predominate). The magnitude of QTL may also depend on the nature of the trait; for example, a study of cold tolerance differences between African and European strains of *D. melanogaster* found six QTL with estimated effect sizes of 5–14% (Svetec *et al.* 2011).

In some cases, overlapping QTL peaks were discovered from different melanic populations (Figure 2 and Table 1), including QTL overlapping *ebony* for both and Ethiopia and Uganda. Rebeiz *et al.* (2009) identified five causative upstream *cis*-regulatory mutations at *ebony* underlying abdominal melanism in our Uganda population, but consistent with that study’s geographic survey, only two of the smaller-effect variants are common in Ethiopia, while the strongest variant is absent. More generally, the high *F_ST_* SNPs for Ethiopia discussed above do not correspond to previously-detected variants underlying pigmentation variation in this species (Bastide *et al.* 2013; Dembeck *et al.* 2015; Endler *et al.* 2016). These results could indicate *cis*-regulatory plasticity of pigmentation (with multiple regulatory elements capable of influencing coloration), or contributions to Ethiopia melanism from other SNPs not highlighted by our population genetic analysis. Different mutations at the same genes drive parallel pigmentation changes in humans (Edwards *et al.* 2010; Yamaguchi *et al.* 2012) and closely-related species of *Peromyscus* mice (Manceau *et al.* 2011; Linnen *et al.* 2013). Our study does not confirm the genetic parallelism or precise molecular basis of pigmentation evolution in *D. melanogaster*, but it sets the stage for functional studies in these melanic populations.

When the same traits were mapped in separate crosses involving the same melanic population, some QTL were identified repeatedly, but overall the results were quite variable (Table 1). At least for QTL with effect sizes below 20%, one potential explanation is simply chance due to incomplete detection power. But in seven cases, stronger QTL (bold in Table 1) were not replicated in another cross for the same population and phenotype, and in light of the high predicted power for their estimated strengths (Pool 2016), these missing QTL seem unlikely to result from type II error. As an example, Figure 3C shows tall QTL peaks near *ebony* and *tan* for an Ethiopia background color cross (EB1). These QTL have estimated effect sizes above 0.33, a strength that resulted in 100% detection power in SIBSAM test simulations (Pool 2016). And yet, neither QTL was detected in our second cross (EB2). In general, results such as these results might stem from causative pigmentation variants that are not fixed differences between dark and light populations. Although the most differentiated SNPs at *ebony*, *tan*, *yellow*, and *black* are simply hypotheses for the mutational targets of selection, it is worth noting that none of these SNPs are fixed in Ethiopia, and only in the case of *tan* is the Ethiopian allele absent from our Zambia sample. EB1, which involved the darker of the two Ethiopian parental strains for background color, had the “Ethiopian allele” at all four focal SNPs discussed above, although no QTL was detected at *black*. EB2 had no QTL detected at *tan* or *ebony*, but it did carry the “Ethiopian allele” at the *tan* SNP and was heterozygous for the *ebony* SNP. Although the presence of an “Ethiopian allele” in a Zambia parental strain could account for QTL absence, this was not the case for either cross at any of these pigmentation genes. These results could point to the contribution of other variants instead of, or in addition to, the maximal *F_ST_* SNPs. Alternatively, unpredictability in QTL mapping results could result from epistatic interactions. In other words, a given variant might have a strong detectable effect or a weak undetectable effect on a pigmentation trait, depending on the genetic backgrounds of the light and dark parental strains used in each mapping experiment.

While QTL often occurred near well-known pigmentation genes (*e.g.*, *ebony*, *tan*, *yellow*, and *black* for Ethiopian background color), none of these loci showed a clear window signal of high genetic differentiation. Instead, we found a recurring pattern of strong frequency differences limited to just one or a few SNPs. For *ebony* in Ethiopia, simulations confirmed that this pattern seems best explained by selection on standing genetic variation. Although the functional significance of our high *F_ST_* variants remains to be confirmed, these narrow intervals of high genetic differentiation support the examination of individual SNPs in scans for local adaptation (*e.g.*, Hübner *et al.* 2013; Reinhardt *et al.* 2014; Bergland *et al.* 2016; Božičević *et al.* 2016; Machado *et al.* 2016; Sedghifar *et al.* 2016).

Our work begins to provide one case study of the genetic architecture of adaptive trait evolution, while offering a foundation for detailed molecular studies to confirm the relevant genes and mutations responsible for parallel melanic evolution in *D. melanogaster*. These results provide one partial set of answers to the basic questions posed at the beginning of this article concerning the genetics of adaptation. However, it will be important to conduct similar studies of different adaptive traits in different organisms to assess the generality of our findings.

Overall, our work highlights significant challenges to mapping the genetic basis of adaptive population differences. Even when relatively large effect loci are present, their detection may depend upon the specific genetic backgrounds used for QTL mapping and genetic targets of local adaptation may not be detected in typical population genetic scans. Nevertheless, continued advances in the analysis of genomic data and molecular methods for confirming adaptive variants have the potential to expand our understanding of the nuanced process of adaptive trait evolution.
